# The Power of the Lorentz Quantum Computer

**DOI:** 10.3390/e28030266

**Published:** 2026-02-28

**Authors:** Qi Zhang, Biao Wu

**Affiliations:** 1 College of Science, Liaoning Petrochemical University, Fushun 113001, China; zhangqi0446@sina.com; 2 Liaoning Provincial Key Laboratory of Novel Micro-Nano Functional Materials, Fushun 113001, China; 3 International Center for Quantum Materials, Peking University, Beijing 100871, China; 4 Wilczek Quantum Center, Shanghai Institute for Advanced Studies, University of Science and Technology of China, Shanghai 201315, China; 5 Hefei National Laboratory, Hefei 230088, China

**Keywords:** Lorentz quantum computer, hyperbolic bit, maximum independent set

## Abstract

We analyze the power of the recently proposed Lorentz quantum computer (LQC), a theoretical model leveraging hyperbolic bits (hybits) governed by complex Lorentz transformations. We define the complexity class BLQP (bounded-error Lorentz quantum polynomial-time) and demonstrate its equivalence to the complexity class $P^{♯P}$ (the class of problems solvable by a deterministic polynomial-time Turing machine with access to a ♯P oracle). LQC algorithms are shown to solve NP-hard problems, such as the maximum independent set (MIS), in polynomial time, thereby placing NP and co-NP within BLQP. Furthermore, we establish that LQC can efficiently simulate quantum computing with postselection (PostBQP), while the reverse is not possible, highlighting LQC’s unique “super-postselection” capability. By proving BLQP $=P^{♯P}$, we situate the entire polynomial hierarchy (PH) within BLQP and reveal profound connections between computational complexity and physical frameworks like Lorentz quantum mechanics. These results underscore LQC’s theoretical superiority over conventional quantum computing models and its potential to redefine boundaries in complexity theory.

## 1. Introduction

Theoretical computing models are fundamentally important in computer science, shaping our understanding of the core principles, boundaries, and possibilities of computing [1,2]. Models like the Turing machine and the quantum Turing machine are physically plausible, serving as abstractions of real-world computers. Conversely, some models are not physically realizable but remain crucial for exploring and clarifying the complexities of computing problems. A prime example is the non-deterministic Turing machine (NDTM), which, despite its theoretical nature, is extensively utilized in the analysis of complexity classes. In particular, the complexity class NP (nondeterministic polynomial time) is defined as the set of decision problems for which a solution can be verified in polynomial time by a deterministic Turing machine. Alternatively, NP can be defined as the set of languages decidable by an NDTM within polynomial time.

**Figure 1:**
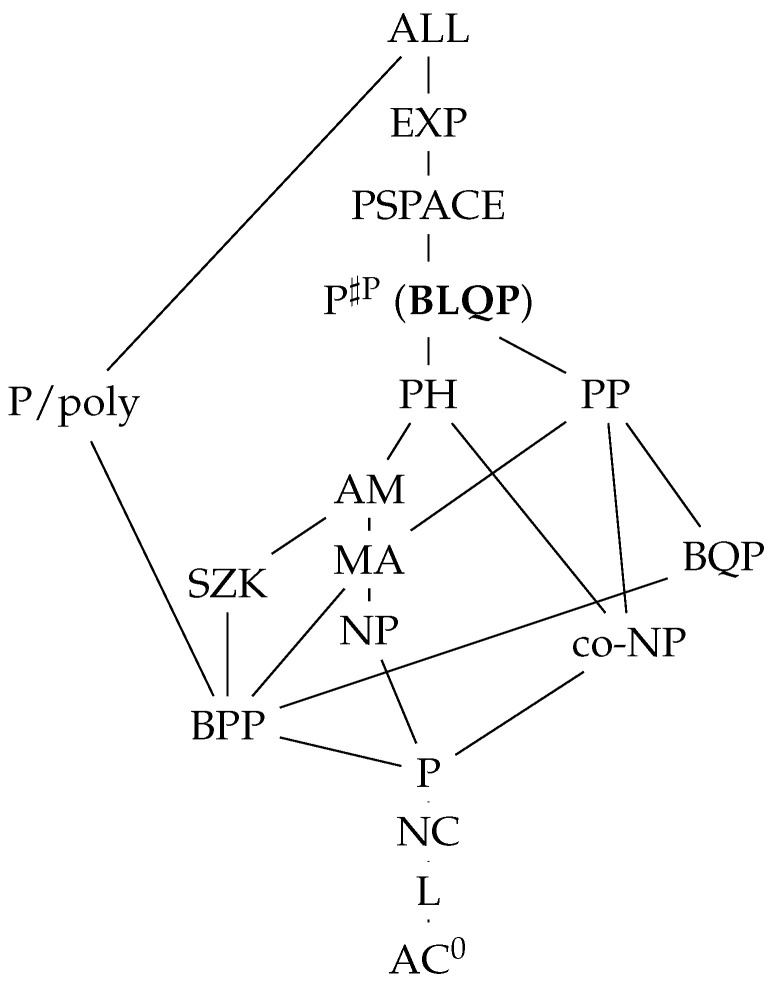
The hierarchy diagram for major complexity classes. For two connecting classes, the class below is included within the class above. BLQP is a complexity class defined for Lorentz quantum computer in parallel to BQP for conventional quantum computer. This diagram without BLQP can be found at www.complexityzoo.com.

A quantum computer with postselection is another theoretical model that is not physically sound because “the ability to postselect on a measurement yielding a specific outcome” is beyond the basic principle of quantum mechanics [3,4]. Nevertheless, this model is theoretically valuable, illuminating the complexity class PP (probabilistic polynomial-time)—see Figure 1—and uncovering connections between quantum mechanics’ core principles and the constraints of quantum computing [4]. The class PP contains decision problems solvable by a probabilistic Turing machine in polynomial time with an error probability less than 1/2 (but not necessarily bounded away from 1/2 by an inverse polynomial).

The theoretical framework for the Lorentz Quantum Computer (LQC) has recently been introduced [5], featuring the innovative concept of the hyperbolic bit (hybit), which evolves via complex Lorentz transformations. This framework draws inspiration from the dynamics of bosonic Bogoliubov quasi-particles, with significant references including works such as [6,7]. Despite its theoretical appeal, the current model lacks practical feasibility. Notably, recent studies on non-Hermitian quantum systems have shown a close connection between non-unitary evolution and postselection, further underscoring the theoretical relevance of models like LQC in exploring the limits of quantum computation [8].

The concept of an indefinite inner product, originally proposed by Dirac, has been further developed to tackle convergence issues in quantized field theories. This concept is integral to the λ-limiting process, which considers the representation of physical observables through self-adjoint operators, as opposed to Hermitian ones [9,10]. There is optimism that the practicability of this model will enhance as future research achieves a unified theory that incorporates both quantum mechanics and gravity under the Lorentz quantum mechanics framework.

Despite its current constraints, the LQC model remains a subject of substantial theoretical interest. It has been highlighted in [5] that LQC potentially offers exponential acceleration in algorithms such as the Grover search algorithm [11], surpassing the capabilities of conventional quantum computers.

In this study, we systematically explore the capabilities of LQC. By analogy with the BQP (bounded-error quantum polynomial-time) complexity class for quantum computing [12], which contains decision problems solvable by a quantum computer in polynomial time with bounded error, we propose a new complexity class for LQC, termed BLQP (bounded-error Lorentz quantum polynomial-time), encompassing problems solvable by LQC within polynomial time and with bounded errors. As the conventional quantum computer is a special case of LQC, it is evident that BQP is a subset of BLQP. We showcase LQC circuits capable of polynomially solving the NP-hard problem of finding the maximum independent set, thereby situating both NP and co-NP as subsets of BLQP. Our research further presents LQC algorithms that efficiently handle problems solvable in polynomial time within the complexity classes PP and hence $P^{♯P}$, establishing its equivalence to the $P^{♯P}$ complexity class. $P^{♯P}$ refers to the class of problems solvable in polynomial time given access to a ♯P oracle, where a ♯P oracle is one that, when provided with an exponential number of instances of a problem in P (i.e., decision problems with true/false outputs), can return in a single step the number of those instances for which the answer is true.

A detailed comparison between LQC and quantum computing with postselection [4] is presented, highlighting LQC’s efficiency in simulating postselection and introducing a unique LQC capability termed super-postselection, which quantum computing with postselection cannot mimic. Consequently, the complexity class PostBQP, designated for quantum computing with postselection, is encompassed within BLQP.

We proceed with a concise review of LQC fundamentals and introduce two pivotal logic gates, the CV gate and CCV gate, essential for our effective algorithms tackling problems in NP, PP, and $P^{♯P}$ classes, illustrating LQC’s substantial edge over traditional quantum computing. The discussion concludes by contrasting LQC with quantum computing with postselection, further elucidating their relationship.

## 2. Theoretical Model of Lorentz Quantum Computer

In the referenced paper [5], a Lorentz quantum computer (LQC) model is detailed, drawing from the principles of Lorentz quantum mechanics [10], an extension of the Bogoliubov–de Gennes equation, which describes bosonic Bogoliubov quasiparticle dynamics. A distinctive feature of these systems is their dual excitation branches, with only the bosonic Bogoliubov quasiparticles considered physically observable, while the negative energy counterpart is deemed unobservable [6]. LQC capitalizes on this characteristic by introducing hyperbolic bits (or hybits for brevity), where one of its states is observable and the other is not. This concept aligns with prior studies involving systems with indefinite inner products [13], including work by Dirac and Pauli [9,10].

In LQC, information storage involves two types of bits: conventional qubits and unique hybits. Qubits function as they do in standard quantum computing, obeying unitary transformations, while hybits are exclusive to LQC and undergo complex Lorentz evolution under gate operations. The state of a hybit, denoted as |ψ), is expressed as

(1) $$\left|\psi \right)=a|0)+b|1)=\left(\begin{array}{c} a\\ b \end{array}\right),$$

where |0) and |1) are the computational bases satisfying

(2) $$\left(0\right|\sigma_{z}\left|0\right)=1\;,\;\;(1|\sigma_{z}\left|1\right)=-1\;,\;\;(1|\sigma_{z}\left|0\right)=0\;.$$

Here, $\sigma_{z}=\mathrm{diag}\left\{1,-1\right\}$ is the familiar Pauli *z* matrix. In the following notation, | ) denotes the state of a hybit, while | 〉 denotes the state of conventional qubit. Hybits |ψ) evolve according to Lorentz quantum mechanics, maintaining a constant indefinite inner product over time

(3) $$\frac{d}{dt}\left(\psi \right|\sigma_{z}\left|\psi \right)=0\;.$$

All the logic gates acting on a hybit induce Lorentz transformations, which preserve the indefinite inner product. For example, if a hybit is in the state of $\left|\psi \right)={\left(a,b\right)}^{T}$, after a gate operation *G*, it becomes $G|\psi )={\left(a^{\prime },b^{\prime }\right)}^{T}$, then we must have $|a^{\prime }{|}^{2}-|b^{\prime }{|}^{2}={\left|a\right|}^{2}-{\left|b\right|}^{2}$. An important consequence is that there is no $\sigma_{x}$ operation that flips between the hybit states |0) and |1), because $(0|\sigma_{z}|0)=1$ and $(1|\sigma_{z}|1)=-1$.

Inherited from Lorentz quantum mechanics, for the two basis of a hybit, only |0) is observable, and |1) is unobservable. This is a fundamental and crucial property of the hybit; as we will see later, the power of LQC is largely derived from this feature. The extension to a multibit scenario is straightforward (for a full elaboration, see Ref. [5]).

Consider an LQC consisting of $N_{q}$ qubits and $N_{h}$ hybits. Its state |Φ) can be expressed in the computational basis as

(4) $$\left|\Phi \right)=\sum_{j=1}^{2^{N_{q}+N_{h}}}a_{j}\left|\psi_{j}\right)\;,$$

where

(5) $$\begin{array}{ccc} |\psi_{j}) & = & |q_{1}\rangle ⊗|q_{2}\rangle \cdots ⊗|q_{i}\rangle \cdots ⊗|q_{N_{q}}\rangle ⊗|h_{1})⊗|h_{2})\cdots ⊗|h_{i})\cdots ⊗|h_{N_{h}})\\ & = & |q_{1},q_{2}\cdots q_{i}\cdots q_{N_{q}};h_{1},h_{2}\cdots h_{i}\cdots h_{N_{h}})\;, \end{array}$$

where $q_{i}$ and $h_{i}$ take values of either 0 or 1.

As long as $N_{h}\neq 0$, the LQC evolves according to the Lorentz transformation. Therefore, a multibit state must satisfy the indefinite inner product condition if at least one bit is a hybit. Consequently, a pure state of a system containing both qubits and hybits must be Lorentzian and is represented in the form |).

It is important to note that if a term $|\psi_{j})$ contains at least one |1), it is not observable. For example, |1,0⋯,0;1,0,⋯,0) is not observable. Also note that if $N_{h}=0$, an LQC is reduced to a conventional quantum computer. In other words, a quantum computer is a special case of a Lorentz computer.

It has been established [5] that the universal gates of an LQC consist of both single-bit gates and two-bit gates in three distinct sets: {H,T}, {τ,T}, and $\left\{\Lambda_{1}^{qq}\left(\sigma_{z}\right),\Lambda_{1}^{qh}\left(\sigma_{z}\right),\Lambda_{1}^{hq}\left(\sigma_{z}\right),\Lambda_{1}^{hh}\left(\sigma_{z}\right)\right\}$, where the subscript 1 indicates that there is one control bit. The first set {H,T} is the Hadamard gate *H* and the π/8 gate *T*,

(6) $$H=\frac{1}{\sqrt{2}}\left(\sigma_{x}+\sigma_{z}\right),\;T=e^{-i\frac{\pi }{8}}\left(\begin{array}{cc} e^{i\pi /8} & 0\\ 0 & e^{-i\pi /8} \end{array}\right)\;.$$

**Figure 2:**
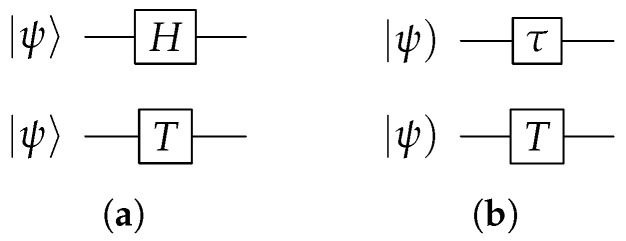
(**a**) Single qubit gates *H* and *T*; (**b**) single hybit gates τ and *T*.

They are applicable to single qubits, and their combined application can approximate any single qubit transformation with arbitrary precision. They are represented in circuits by the symbols in Figure 2a. 

The second set operates on single hybits and consists of the π/8 gate *T* and the τ gate. The *T* gate has the same matrix form as the *T* gate for qubits, and the matrix form of the τ gate is

(7) $$\begin{array}{c} \tau =\sqrt{2}\sigma_{z}+i\sigma_{x}=\left(\begin{array}{cc} \sqrt{2} & i\\ i & -\sqrt{2} \end{array}\right)\;. \end{array}$$

These two gates are applicable to single hybits. Their symbols in circuits are shown in Figure 2b. It is noteworthy that the operator *H* is unitary and τ is Lorentzian, while *T* is both unitary and Lorentzian.

**Figure 3:**
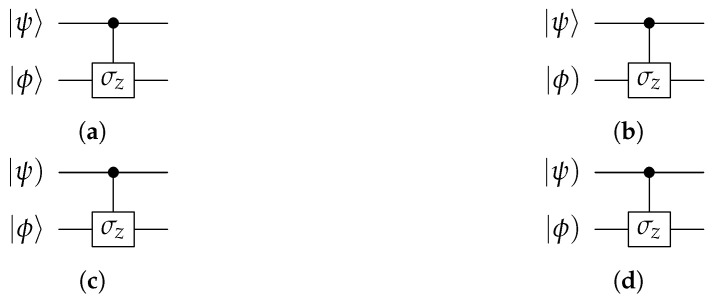
Four different controlled-$\sigma_{z}$ gates. (**a**) Circuit for $\Lambda_{1}^{qq}\left(\sigma_{z}\right)$. (**b**) Circuit for $\Lambda_{1}^{qh}\left(\sigma_{z}\right)$. (**c**) Circuit for $\Lambda_{1}^{hq}\left(\sigma_{z}\right)$. (**d**) Circuit for $\Lambda_{1}^{hh}\left(\sigma_{z}\right)$.

The logical gates in the final set, denoted as $\Lambda_{1}^{qq}\left(\sigma_{z}\right)$, $\Lambda_{1}^{qh}\left(\sigma_{z}\right)$, $\Lambda_{1}^{hq}\left(\sigma_{z}\right)$ and $\Lambda_{1}^{hh}\left(\sigma_{z}\right)$, represent four variations of controlled-$\sigma_{z}$ operators. These variations differ themselves by the types of the control and target bits as indicated by the superscripts: *q* for qubit and *h* for hybit. The corresponding circuits are illustrated in Figure 3. Notably, we have chosen the controlled-$\sigma_{z}$ gate over the controlled-NOT (CNOT) gate, which is a more common choice in quantum computing. This decision is motivated by the fact that the CNOT gate is a unitary transformation, which does not hold for a hybit. In contrast, the controlled-$\sigma_{z}$ gate is both unitary and Lorentzian.

Note that the gates $\Lambda_{1}^{qq}\left(\sigma_{z}\right)$, $\Lambda_{1}^{qh}\left(\sigma_{z}\right)$, $\Lambda_{1}^{hq}\left(\sigma_{z}\right)$ and $\Lambda_{1}^{hh}\left(\sigma_{z}\right)$ are denoted in Ref. [5] as $\Lambda_{1}^{qq}\left(\sigma_{z}\right)$, $\Lambda_{1}^{ql}\left(\sigma_{z}\right)$, $\Lambda_{1}^{lq}\left(\sigma_{z}\right)$ and $\Lambda_{1}^{ll}\left(\sigma_{z}\right)$, respectively. The superscript *l* is replaced by *h* in this paper to avoid confusion.

It has been established [5] that any Lorentz transformation of the state |Φ) in Equation (4) can be realized by a combination of the gate sets {H,T}, {τ,T}, and $\left\{\Lambda_{1}^{qq}\left(\sigma_{z}\right),\Lambda_{1}^{qh}\left(\sigma_{z}\right),\Lambda_{1}^{hq}\left(\sigma_{z}\right),\Lambda_{1}^{hh}\left(\sigma_{z}\right)\right\}$.

**Figure 4:**
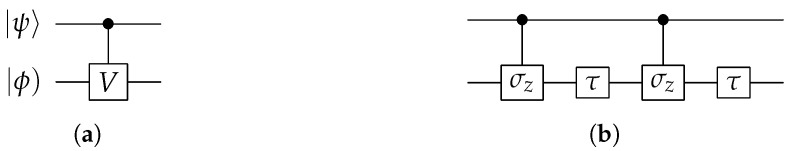
(**a**) Two-bit logical CV gate; (**b**) a simple way to realize CV using the controlled-$\sigma_{z}$ gate and the τ gate for $\chi =2\ln(\sqrt{2}+1)$.

The following sections present powerful LQC algorithms for solving difficult problems. In these algorithms, one two-bit control gate is used repeatedly. It is the controlled-*V* gate $\Lambda_{1}^{qh}\left(V\right)$, and we will denote it as CV. Its circuit is shown in Figure 4a, where the control bit is a qubit, and the target bit is a hybit. If the qubit is in the state of |0〉, nothing happens; if it is in the state of |1〉, the hybit undergoes a complex Lorentz transformation

(8) $$V=\left(\begin{array}{cc} \cosh\chi & -i\sinh\chi \\ i\sinh\chi & \cosh\chi \end{array}\right),$$

where χ is a positive constant. The transformation *V* is actually a hyperbolic rotation: for a positive integer *r*, we have

(9) $$V^{r}=\left(\begin{array}{cc} \cosh r\chi & -i\sinh r\chi \\ i\sinh r\chi & \cosh r\chi \end{array}\right)\;.$$

For $\chi =2\ln(\sqrt{2}+1)$, as shown in Figure 4b, the CV gate can be realized with two τ gates and two controlled-$\sigma_{z}$ gates.

**Figure 5:**
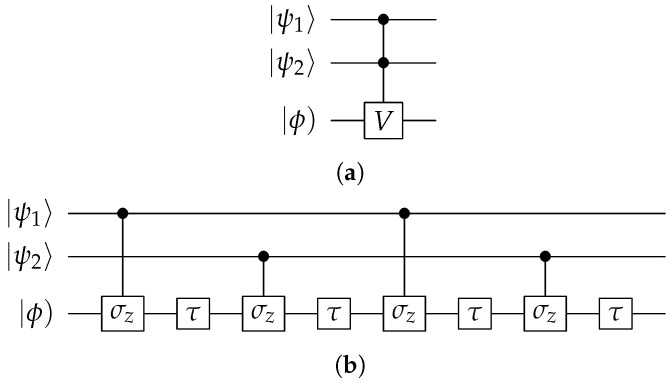
(**a**) Three-bit logical CCV gate; (**b**) the circuit that implements the CCV gate with four τ gates and four controlled-$\sigma_{z}$ gates for $\chi =4\ln(\sqrt{2}+1)$.

We also often use a three-bit logic gate as shown in Figure 5a, where the two qubits are control bits, and the hybit is the target bit. Only when both qubits are in state |1〉 does the target hybit undergo the Lorentz transformation *V*; otherwise, nothing happens. We call this a CCV gate, which can be realized with a circuit in Figure 5b. This circuit consists of four τ gates and four controlled-$\sigma_{z}$ gates for $\chi =4\ln(\sqrt{2}+1)$.

**Physical Interpretation of CV and CCV Gates.** The CV and CCV gates exploit the unique property of hybits: state |1) is unobservable. By applying a Lorentz transformation *V* conditioned on the control qubit(s), these gates amplify the amplitude of the target hybit’s |0) component, effectively enhancing the weight of the corresponding computational basis states. This amplification is achieved without violating the indefinite inner-product preservation, and it allows the LQC to selectively emphasize specific branches of the superposition. In physical terms, this process resembles an exponential squeezing of probability amplitudes, enabled by the non-unitary but norm-preserving (under the indefinite metric) evolution.

The CV and CCV gates are at the heart of LQC’s computational power, since the Lorentz transformation in Equation (8) has the ability to amplify the components of a hybit state without limit. Consider a system of a qubit and a hybit that is in the state of

(10) $$|\phi_{0})=\frac{\sqrt{2}}{2}\left[|0\rangle +|1\rangle \right]⊗\left|0\right)\;.$$

After the application of a CV gate, the state becomes

(11) $$\frac{\sqrt{2}}{2}|0\rangle ⊗\left|0\right)+\frac{\sqrt{2}}{2}|1\rangle ⊗\left[\cosh\chi |0)+i\sinh\chi |1)\right]$$

As mentioned before, state |1) for a hybit is unobservable so that we only need to consider the two terms that contain |0), which are

(12) $$|\phi_{1})=\frac{\sqrt{2}}{2}\left[|0\rangle +\cosh\chi |1\rangle \right]⊗\left|0\right)\;.$$

Compared to state $|\phi_{0})$, it is clear that the weight of state |1〉 has increased in both absolute and relative terms. As we will see in the following sections, this capability of the gate CV gives the LQC a significant computational advantage over the conventional quantum computer. The gate CCV has a similar ability to selectively amplify. With this capacity of amplification in mind, we introduce the formal definition of BLQP, a computational complexity class of languages related to the LQC.

*Definition of BLQP*. For a language L within BLQP, there exists a uniform family of quantum circuits, denoted as ${\left\{C_{n}\right\}}_{n\geq 1}$, where each circuit is of polynomial size. These circuits employ qubits and hybits, as well as unitary and Lorentzian gates, and they allow measurements, after which no further quantum gates can be applied. Given an input of length *n* and specific initial states for the work qubits and hybits, the circuit $C_{n}$ operates for polynomial time in *n* and then halts. For ω∈L, the probability of obtaining an accepting state is higher than 2/3. Conversely, for ω∉L, this probability is less than 1/3.

The criterion for an accepting state can involve either all bits in $C_{n}$ or a single qubit. For instance, a specific qubit, referred to as the “Y qubit”, can be used for this purpose. An accepting state is defined as

(13) $$\left|\mathrm{accept})=\right|\Psi_{1}\rangle ⊗|00\ldots 0)⊗|1_{Y}\rangle ,$$

while a rejecting state is

(14) $$\left|\mathrm{reject})=\right|\Psi_{2}\rangle ⊗|00\ldots 0)⊗|0_{Y}\rangle ,$$

where $|\Psi_{1}\rangle$ and $|\Psi_{2}\rangle$ (with $\langle \Psi_{1}|\Psi_{1}\rangle =\langle \Psi_{2}|\Psi_{2}\rangle =1$) represent the states of all qubits determined by the circuit’s output. The term |00…0) indicates that all hybits are in state |0), where |0) is detectable, and |1) is undetectable. The subscript *Y* in $|1_{Y}\rangle$ and $|0_{Y}\rangle$ denotes the state of the “Y qubit”.

According to the properties of hybits, for ω∈L, the output of $C_{n}$ will be of the form

(15) $$\left|\psi \right)=c_{\mathrm{yes}}\left|\mathrm{yes}\right)+c_{\mathrm{no}}\left|\mathrm{no}\right)+c_{3}\left|\Psi_{3}\right),$$

where $\frac{c_{\mathrm{yes}}^{2}}{c_{\mathrm{yes}}^{2}+c_{\mathrm{no}}^{2}}>2/3$. Here, $|\Psi_{3})$ represents the overall state associated with all bits in the circuit when at least one hybit is in the undetectable state |1).

Similarly, for an input ω∉L of length *n*, the output of $C_{n}$ will also be in the form of the expression above, but with $\frac{c_{\mathrm{yes}}^{2}}{c_{\mathrm{yes}}^{2}+c_{\mathrm{no}}^{2}}<1/3$.

For convenience, this error probability is often expressed as an exponentially small quantity rather than using 1/3.

## 3. LQC Algorithms for the Maximum Independent Set

In this section, we present an example that demonstrates the LQC algorithm, specifically by solving the maximum independent set (MIS) problem in polynomial time. This example serves as an initial demonstration of LQC’s capabilities and highlights the subtle differences between an LQC and quantum computers with postselection. Given that MIS is NP-hard [14], this directly implies that an LQC can polynomially solve all problems within the NP and co-NP classes. It is noteworthy that MIS has not yet been proven to belong to PP. Therefore, if we prove that MIS belongs to BLQP, it would imply that we currently cannot demonstrate that BLQP is contained within PP.

For a graph G(n,m) with *n* vertices and *m* edges, an independent set (IS) is a subset of the vertices that are not directly connected by edges. The MIS are those with the largest number of vertices among all ISs. For a given graph, finding its MIS is difficult on a classical computer and it is an NP-hard problem [14]. A recently proposed quantum algorithm shows promising signs of exponential speedup [15,16]; however, there is no rigorous proof or very convincing numerical evidence. Here, we present an LQC algorithm that can solve MIS problems in polynomial time.

To design the algorithm for a given graph G(n,m), we assign a Boolean variable to each vertex, $x_{1},x_{2},\cdots ,x_{n}$. As a result, a subset of the vertices is represented by an integer *x* in its *n*-digit binary form: if its *i*th digit $x_{i}=1$, then the *i*th vertex is in the subset; if $x_{i}=0$, then it is not. If *x* is an IS, then its $x_{i}$ and $x_{j}$ cannot both be 1 simultaneously if the two vertices $x_{i}$ and $x_{j}$ are connected by an edge.

For an LQC algorithm, we use *n* work qubits to represent the *n* vertices. Their $N=2^{n}$ possible states |00…0〉, |00…1〉, …, |11…1〉 naturally represent all the subsets of vertices. That is, a basis vector |x〉 corresponds to the subset *x* where the integer *x* is in its binary form. The goal is to find the target state |M〉 that corresponds to MIS out of the $N=2^{n}$ possible states.

**Figure 6:**
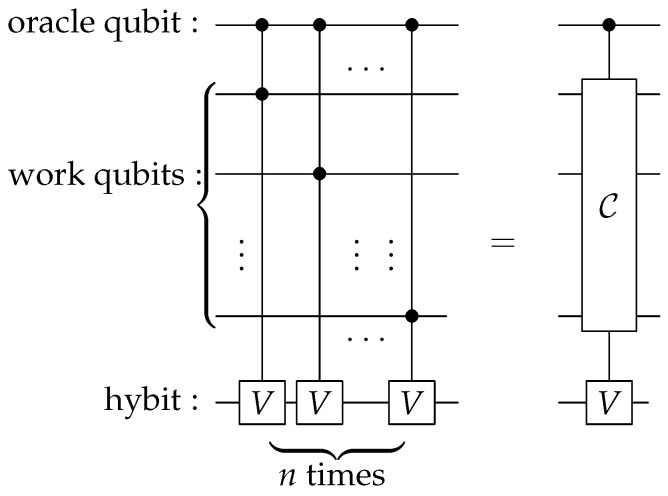
LQC circuit for the operation *Q* that is capable of counting the number of ones that are in the basis state |x〉. It consists of *n* CCV gates. The right circuit is a short-hand representation of the left circuit.

In our algorithm for MIS problems, we add an oracle qubit and a hybit on top of the *n* work qubit in the computation circuit. The main part of our algorithm is shown in Figure 6, which consists of *n* CCV gates. To see its functionality, let us consider two basis states |x〉 and |y〉: *x* is not an IS and *y* is an IS. To distinguish them, we entangle them with the oracle qubit and prepare the following initial state

(16) $$|\phi_{0})=\frac{1}{\sqrt{2}}\left(|x\rangle ⊗|0_{o}\rangle +|y\rangle ⊗|1_{o}\rangle \right)⊗\left|0\right)\;.$$

The *Q* operation shown in Figure 6 consists of *n* CCV gates. After its application, the state at the output is

(17) $$\begin{array}{cc} |\phi_{1})= & Q|\phi_{0})=\frac{1}{\sqrt{2}}\left[|x\rangle ⊗|0_{o}\rangle ⊗\left|0\right)+\cosh\left(m_{y}\chi \right)|y\rangle ⊗|1_{o}\rangle ⊗\left|0\right)+i\sinh\left(m_{y}\chi \right)|y\rangle ⊗|1_{o}\rangle ⊗\left|1\right)\right]\;, \end{array}$$

where $m_{y}$ is the number of ones in the binary form of *y*. As emphasized in the last section, the hybit state |1) is unobservable. So, the above state effectively has only the first two terms. As a result, the weight of the state |y〉 is enhanced by a factor of $\cosh(m_{y}\chi )$, which is determined by $m_{y}$, the number of ones in *y*. This means that the circuit in Figure 6 effectively has the ability to count the number of ones in *y*, which for the graph is the number of vertices in the subset *y*.

To achieve an entangled state similar to $|\phi_{0})$ in Equation (16), we use the following oracle

(18) $$O_{\mathrm{IS}}=\left(I-P_{\mathrm{IS}}\right)⊗I_{o}+P_{\mathrm{IS}}⊗(|0_{o}\rangle \langle 1_{o}\left|+\right|1_{o}\rangle \langle 0_{o}|)\;,$$

where $I_{o}$ is the identity matrix for the oracle qubit, and $P_{\mathrm{IS}}$ is a projection onto the sub-Hilbert space spanned by all possible solutions |j〉 of IS,

(19) $$P_{\mathrm{IS}}=\sum_{x\in \mathrm{IS}}|x\rangle \langle x|\;.$$

The quantum oracle $O_{\mathrm{IS}}$ is similar to the one used in the Grover algorithm [12], and it evaluates whether a subset *x* is an IS in polynomial time.

**Figure 7:**
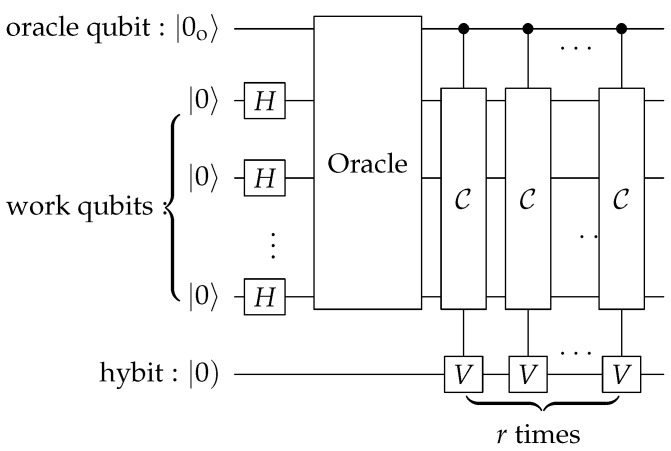
Circuit of an LQC algorithm for solving MIS problems in polynomial time. The big box represents the oracle that implements the operator (18). As explained in the text, *r* is proportional to *n*.

The circuit of our algorithm is shown in Figure 7. The initial state of the whole system, including the *n* work qubits, one oracle qubit and one hybit, is set to be $\left|00\ldots 0\rangle ⊗\right|0_{o}\rangle ⊗|0)$. The algorithm then proceeds as follows:

(i) Apply Hadamard gates to all work qubits;
(ii) Apply the oracle $O_{\mathrm{IS}}$;
(iii) Apply the *Q* operation *r* times;
(iv) Measure the oracle qubit and the hybit.

After the step (i), the state becomes

(20) $$\begin{array}{c} |\Psi_{0})=|\Phi_{0}\rangle ⊗|0_{o}\rangle ⊗|0)=\frac{1}{\sqrt{N}}\left(\sum_{x=0}^{2^{n}-1}|x\rangle \right)⊗|0_{o}\rangle ⊗|0)\;. \end{array}$$

With the oracle operation in the step (ii), we have

(21) $$\begin{array}{cc} |\Psi_{1})= & O_{\mathrm{IS}}|\Phi_{0}\rangle ⊗|0_{o}\rangle ⊗|0)=\frac{1}{\sqrt{N}}\left(\sum_{j\notin \mathrm{IS}}\left|j\rangle ⊗\right|0_{o}\rangle +\sum_{x\in \mathrm{IS}}\left|x\rangle ⊗\right|1_{o}\rangle \right)⊗\left|0\right)\;. \end{array}$$

After the step (iii), we obtain

(22) $$\begin{array}{cc} |\Psi_{2})=Q^{r}\left|\Psi_{1}\right) & =\frac{1}{\sqrt{N}}\left(\sum_{j\notin \mathrm{IS}}|j\rangle ⊗|0_{o}\rangle +\sum_{x\in \mathrm{IS}}\cosh\left(m_{x}r\chi \right)|x\rangle ⊗|1_{o}\rangle \right)⊗\left|0\right)\\ \; & +\frac{1}{\sqrt{N}}\left(\sum_{x\in \mathrm{IS}}i\sinh\left(m_{x}r\chi \right)|x\rangle ⊗|1_{o}\rangle \right)⊗\left|1\right), \end{array}$$

where $m_{x}$ is the number of ones in the binary form of *x* or, equivalently, the number of vertices in the IS *x*. As mentioned in the last section, the hybit state |1) is not observable. So, the probability *P* of getting the MIS after the measurement is given by

(23) $$P=\frac{N_{\mathrm{MIS}}\cosh^{2}\left(Mr\chi \right)}{N-N_{\mathrm{IS}}+\sum_{x\in \mathrm{IS}}\cosh^{2}\left(m_{x}r\chi \right)},$$

where *M* is the number of vertices in the MIS, $N_{\mathrm{IS}}$ is the number of ISs, and $N_{\mathrm{MIS}}$ is the number of MIS. It is obvious that we have

(24) $$\begin{array}{c} P>\frac{N_{\mathrm{MIS}}\cosh^{2}\left(Mr\chi \right)}{\left(N-N_{\mathrm{MIS}}\right)\cosh^{2}\left(\left(M-1\right)r\chi \right)+N_{\mathrm{MIS}}\cosh^{2}\left(Mr\chi \right)}\approx \frac{N_{\mathrm{MIS}}e^{2r\chi }}{N-N_{\mathrm{MIS}}+N_{\mathrm{MIS}}e^{2r\chi }}. \end{array}$$

It is clear that P≈1 when $r\approx \frac{1}{\chi }\ln N\propto n$. Since each execution of *Q* involves *n* CCV gates, the time complexity of our algorithm is $O\left(n\ln N\right)∼O\left(n^{2}\right)$.

The definition of BLQP is inherently linked to decision problems. By trivially extending the circuit, we can transform the problem into a decision problem. The input for the decision algorithm is of the form “G(n,m) (a graph with *n* vertices and *m* edges) + *S* (a subset of vertices in *G*)”, and the output is of the form shown in Equation (15),

(25) $$\left|\psi \right)=c_{\mathrm{yes}}\left|\mathrm{yes}\right)+c_{\mathrm{no}}\left|\mathrm{no}\right)+c_{3}\left|\Psi_{3}\right),$$

where $\frac{c_{\mathrm{yes}}^{2}}{c_{\mathrm{yes}}^{2}+c_{\mathrm{no}}^{2}}>2/3$ if *S* forms an MIS in *G*, and $\frac{c_{\mathrm{yes}}^{2}}{c_{\mathrm{yes}}^{2}+c_{\mathrm{no}}^{2}}<1/3$ if *S* does not form an MIS. $|\Psi_{3})$ represents the overall state associated with all bits in the circuit when at least one hybit is in the undetectable state |1).

This extension to the decision circuit can be easily achieved by adding an additional oracle to the original circuit, as shown by the small box in Figure 7. The portion of input G(n,m) is used for the initial circuit in Figure 7, while the portion of *S* serves as the input for the additional oracle. An MIS can be obtained from Figure 7, and hence, we can efficiently calculate the size of the MIS. By checking whether *S* is an IS and whether the size of *S* matches the calculated value, we can efficiently decide whether *S* is an MIS.

It is clear that by extending the circuit above for this decision problem, ${|c_{\mathrm{yes}}|}^{2}$ will be nearly 1 if *S* is an MIS, and nearly 0 if *S* is not. Since the LQC can solve the MIS problem, which is NP-hard, in polynomial time, it follows that both NP and co-NP are subsets of BLQP.

In fact, the MIS problem is also in the $P^{\mathrm{NP}}$ complexity class belonging to PH (polynomial hierarchy). The *k*-IS, which involves finding an independent set of *k* vertices, falls within NP. We can submit *n* non-adaptive queries to the NP-oracle (or SAT-oracle since the SAT problem is NP-complete) for 0-IS, 1-IS, all the way up to *n*-IS solutions. By determining the maximum value of $k_{\mathrm{MAX}}$ that yields a positive result from the oracle, we derive the solution for MIS. Thus, MIS is in the class $P^{\|\mathrm{NP}}$​​ [17,18].

## 4. $P^{♯P}$ = BLQP

Starting from this section, we will begin our main discussion by proving that $P^{♯P}$ = BLQP. First, we present an algorithm for a PP-complete problem, named MAJSAT. Next, we demonstrate how this algorithm can be extended to solve problems in the $P^{♯P}$ class in polynomial time, thereby establishing that $P^{♯P}\subseteq$BLQP. Finally, we provide two distinct proofs showing that $\mathrm{BLQP}\subseteq P^{♯P}$, thereby proving that $P^{♯P}$ = BLQP.

### 4.1. LQC Algorithm for PP

Instead of considering the class of PP problems in general, we focus on a PP-complete problem, MAJSAT, and discuss the approach to solving it with an LQC. For a given Boolean expression $f(x_{1},x_{2},\cdots ,x_{n})$ of *n* Boolean variables, the problem of MAJSAT is to determine whether major assignments of Boolean variables satisfy f=1. To understand why MAJSAT is PP-complete, please consult some textbooks on computational complexity theory, for example, Ref. [1]. For a given Boolean formula *f*, we let *s* be the number of assignments of *n* Boolean variables satisfying f=1. The problem of MAJSAT is to determine whether $s>2^{n-1}$.

**Figure 8:**
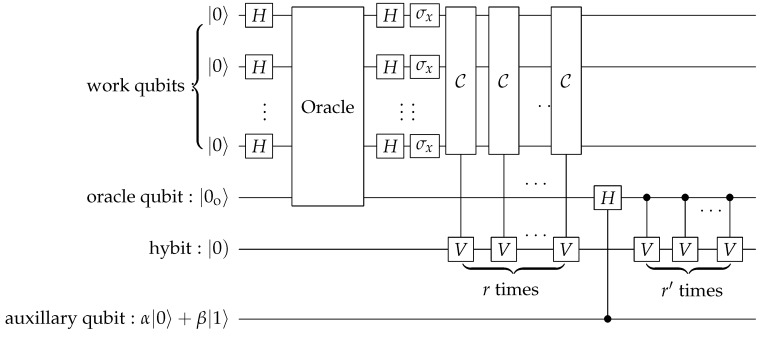
Circuit of an LQC algorithm for solving MAJSAT, which is PP-complete. The auxiliary qubit is initialized in α|0〉+β|1〉 with $\beta /\alpha =2^{i}$, where *i* is an integer ranging from −n to *n*.

The LQC circuit for solving MAJSAT is depicted in Figure 8, where an auxiliary qubit is used together with *n* work qubit, an oracle qubit and a hybit. The quantum oracle used here is similar to the one in Figure 7 and has the ability to evaluate *f* in parallel in polynomial time. We continue to use binary notation, i.e., in a state |x〉 of *n* work qubit, the integer *x* is understood in its binary form. The algorithmic steps shown in Figure 8 are as follows:

(i) Initialize all bits to either |0〉 or |0) except the auxiliary qubit, which is set to the state of $|\varphi_{\beta /\alpha }\rangle =\alpha |0\rangle +\beta |1\rangle$, where α and β are real and positive.
(ii) Apply Hadamard gates to each of the work qubits,
  (26) $$|\Psi_{\mathrm{ii}})=|\Phi_{0}\rangle ⊗|0_{o}\rangle ⊗|0)⊗|\varphi_{\beta /\alpha }\rangle =\frac{1}{\sqrt{N}}\left(\sum_{j=0}^{2^{n}-1}|j\rangle \right)⊗|0_{o}\rangle ⊗|0)⊗|\varphi_{\beta /\alpha }\rangle .$$
(iii) Apply the oracle operator *O* to the state vector,
  (27) $$O=\left(I-P_{s}\right)⊗I_{o}+P_{s}⊗(|0_{o}\rangle \langle 1_{o}\left|+\right|1_{o}\rangle \langle 0_{o}|)\;,$$
  where $I_{o}$ is the identity matrix for the oracle qubit, and $P_{s}$ is a projection onto the sub-Hilbert space spanned by all possible solutions |j〉 of f=1
  (28) $$P_{s}=\sum_{j\in \{f=1\}}|j\rangle \langle j|\;.$$
  followed by applying Hadamard gates and $\sigma_{x}$-gates to each of the work qubits.
(iv) Apply the *Q* operation (see Figure 6) *r* times without using the oracle qubit as the control bit.
(v) Apply the Hadamard gate to the oracle qubit with the auxiliary qubit as the control bit.
(vi) With the oracle qubit as the control bit and the hybit as the target bit, apply the CV gates $r^{\prime }$ times.
(vii) Measure the auxiliary qubit in the *x*-direction a large number of times, and count the number of one of the two outcomes: 1 and −1. Note that one needs to repeat steps (i)–(vi) for each measurement. The number of measurements will be discussed in the later analysis.
(viii) Repeat the above procedures 2n+1 times, each with a distinct value of $\beta /\alpha =2^{i}$ for the auxiliary qubit, where *i* is an integer ranging from −n to *n* inclusive.

Let us analyze the algorithm to understand why it is capable of solving MAJSAT. After step (iii), the entire system becomes

(29) $$|\Psi_{\mathrm{iii}})=\sum_{x=0}^{N-1}|x\rangle ⊗\left(a_{x}|0_{o}\rangle +b_{x}|1_{o}\rangle \right)⊗\left|0\right)⊗|\varphi_{\beta /\alpha }\rangle \;,$$

where $a_{x}=1$ and $b_{x}=0$ when $f(x_{1},x_{2},\cdots ,x_{n})=0$, and $a_{x}=0$ and $b_{x}=1$ when $f(x_{1},x_{2},\cdots ,x_{n})=1$. With r≈lnN/χ, the subsequent *Q* operations in step (iv) are aimed at effectively isolating the term with |11⋯1〉 among all the possible $N=2^{n}$ terms. Omitting terms with exponentially smaller coefficients and terms with |1) which are unobservable, we have

(30) $$|\Psi_{\mathrm{iv}})\approx |11\cdots 1\rangle ⊗|\phi_{o}\rangle ⊗|0)⊗|\varphi_{\beta /\alpha }\rangle \;,$$

where

(31) $$|\phi_{o}\rangle =\frac{(N-s)|0_{o}\rangle +s|1_{o}\rangle }{\sqrt{{\left(N-s\right)}^{2}+s^{2}}}\;.$$

In this step, the number of satisfying assignments *s* is stored in the coefficients of the oracle qubit state $|\phi_{o}\rangle$. Since the difference between the two coefficients can be exponentially small, in general, one has to measure it exponentially many times in order to tell the difference. Steps (v) and (vi) use the special property of hybit to reduce it to a polynomial number of times.

At step (v), the controlled Hadamard gate mixes up the coefficients of the oracle qubit state and the auxiliary qubit state. At step (vi), the CV gate is applied $r^{\prime }$ times with the oracle qubit as the control bit and the hybit as the target bit. By setting $r^{\prime }\approx \ln N/\chi$, when we measure the oracle qubit, we are almost certain to find it in the state of $|1_{o}\rangle$ and the auxiliary qubit in the state of

(32) $$|\varphi_{\eta }\rangle =\frac{s|0\rangle +\eta \sqrt{1/2}\left(2^{n}-2s\right)|1\rangle }{\sqrt{s^{2}+\left(\eta^{2}/2\right){\left(2^{n}-2s\right)}^{2}}}\;,$$

where η=β/α. The detailed calculation leading to the above equation can be found in Appendix A. As indicated in step (viii), η has 2n+1 possible values, $\eta_{i}=2^{i}$ (i∈[−n,n]).

With $|\varphi_{\eta }\rangle$, it is now possible to determine whether $s>2^{n-1}$ in polynomial time. We regard the auxiliary qubit as a spin and measure it along the *x*-direction for which the two basis vectors are $|\pm \rangle =(|0\rangle \pm |1\rangle )/\sqrt{2}$. When $s\leq 2^{n-1}$, it is easy to show that

(33) $$P_{-}=\left|\langle -|\varphi_{2^{i}}\rangle \right|\leq 1/2.$$

This means that if we measure it a large number of times, the number of outcome −1 will not exceed the number of outcome 1.

When the instance *f* is in MAJSAT, that is, $s>2^{n-1}$, the probability of outcome −1 is

(34) $$P_{-}=\frac{1}{2}+\frac{\sqrt{2}\eta s\left(2s-2^{n}\right)}{2s^{2}+\eta^{2}{\left(2s-2^{n}\right)}^{2}}\;,$$

which is always larger than 1/2. Although $\delta_{p}=P_{-}-1/2$ can be exponentially small for some values of η, we find that for a given value of $\delta_{p}\leq \sqrt{2}/4$, there are some values of η such that, for all possible values of $s>2^{n-1}$, we always have

(35) $$P_{-}\geq \frac{1}{2}+\delta_{p}\;.$$

For convenience, we denote one of such η as $\eta_{a}=2^{m_{a}}$, where $m_{a}$ is an integer between −n and *n*. For detailed analysis, please see Appendix B. Note that $\delta_{p}$ can be set to a small value, but the value is finite and independent of *n*. For a smaller $\delta_{p}$, there are more possible values of η, similar to $\eta_{a}$. Suppose for the special $\eta_{a}$, the auxiliary qubit is measured $N_{\mathrm{PP}}$ times. We find that when

(36) $$N_{\mathrm{PP}}\geq \frac{2\log(\epsilon )}{\log(1-4\delta_{p}^{2})}\;,$$

the probability that the number of measurement results −1 exceeds the number of results 1 for $\eta_{a}$ is P=1−ϵ.

Based on the above analysis, we lay out the procedure to determine whether *f* is in MAJSAT. First, we set $\epsilon =c^{-n}$ with c>1. This makes the value of $N_{\mathrm{PP}}$ becomes a linear function of *n*. Then, for each value of $\eta_{i}=2^{i}$ (i∈[−n,n]), we conduct *n* sets of measurements. Within each set, we perform $N_{\mathrm{PP}}$ measurements. This means that, for any given value of $\eta_{i}=2^{i}$, the auxiliary qubit is measured in the *x*-direction a total of $nN_{\mathrm{PP}}$ times. For each set of $N_{\mathrm{PP}}$ measurements, we designate a result as “success” if the number of results −1 exceeds the number of results 1. For the *n* sets of $N_{\mathrm{PP}}$ measurements, we count the occurrences of the “success” results.

If *f* is in MAJSAT, then the probability of all the results being “success” at $\eta_{a}$ is given by

(37) $$P_{n}={\left(1-c^{-n}\right)}^{n}\;.$$

It is evident that $\lim_{n\to \infty }P_{n}=1$. This means that, for a sufficiently large *n*, there must exist a value of $\eta =\beta /\alpha =2^{i}$ such that the results of all *n* sets of measures are “success”.

However, if *f* is not in MAJSAT, then the probability of having at least one value of η, such that for *n* sets of measures, all the results are “success”, is given by

(38) $$P_{n}\leq 1-{\left(1-2^{-n}\right)}^{2n+1}\;.$$

It is clear that $\lim_{n\to \infty }P_{n}=0$. This implies that for a sufficiently large *n*, it is impossible that all *n* sets of measurements will result in “success” for all ratios of $\eta_{i}=2^{i}$.

With this strategy, we can determine whether an instance *f* belongs to MAJSAT. The time complexity of the entire algorithm is $O(n^{4})$, meaning that it runs in polynomial time.

### 4.2. $P^{♯P}\subseteq$BLQP

According to computational complexity theory, we have $P^{\mathrm{PP}}=P^{♯P}$. Consequently, the LQC algorithm for PP problems can be adapted to efficiently solve problems in ♯P and $P^{♯P}$. In this section, we briefly discuss the algorithm through a specific example, namely, the MAX-*k*-IS problem.

The class ♯P contains problems where the task is to compute the number of accepting paths in a nondeterministic polynomial-time TM. It is a counting version of the class P, which contains decision problems solvable in polynomial time. A ♯P-complete problem is ♯SAT, which is to determine, for a Boolean expression $f(x_{1},x_{2},\ldots ,x_{n})$, the number of assignments of Boolean variables $x_{1},x_{2},\ldots ,x_{n}$ such that f=1. We focus on a $P^{♯P}$ problem called MAX-*k*-IS. For a given graph G(n,m), there are many ISs. Let us denote the set of ISs having *k* vertices as *k*-IS and its size as ♯k-IS. For example, ♯0-IS is one and ♯1-IS is *n*. The problem of MAX-*k*-IS is to determine which ♯k-IS is the largest. MAX-*k*-IS is evidently a $P^{♯P}$ problem. As the *k*-IS is an NP-complete problem, we can query the ♯P-oracle for ♯0-IS, ♯1-IS, up to ♯n-IS, respectively, and compare them to determine which is the largest. We will now show that this problem can be solved by an LQC, using the algorithm for solving PP as shown in Figure 8.

Regarding the graph G(n,m), similar to the previous section, we employ binary notation: $x=x_{1}x_{2}\ldots x_{n}$, where $x_{j}=1$ denotes the selection of the *j*th vertex. Since the *k*-IS is an NP problem, there exists a Boolean expression $f_{k}\left(x_{1},x_{2},\ldots ,x_{n}\right)$ such that $f_{k}=1$ if and only if $x_{1}x_{2}\ldots x_{n}$ forms an independent set containing *k* vertices. To solve this problem with the PP algorithm, we formulate an additional Boolean expression $g_{z}\left(x_{1},x_{2},\ldots ,x_{n}\right)$, where $1\leq z\leq 2^{n}$. This expression evaluates to 1 ($g_{z}=1$) if the string $x=x_{1}x_{2}\ldots x_{n}$, interpreted as a binary number, is less than *z*. For any $1\leq z\leq 2^{n}$, $g_{z}$ can be constructed in a polynomial number of steps; an example is provided in Appendix C.

We next introduce an additional Boolean variable $x_{0}$ and construct a Boolean expression involving n+1 variables,

(39) $$F\left(x_{0},x_{1},\ldots x_{n}\right)=\left(x_{0}\wedge f_{k}\left(x_{1},x_{2},\ldots ,x_{n}\right)\right)\vee \left({\bar{x}}_{0}\wedge g_{z}\left(x_{1},x_{2},\ldots ,x_{n}\right)\right).$$

The expression *F* is true only when either $f_{k}$ or $g_{z}$ is true, and $x_{0}$ here serves as a switch.

With the Boolean expression (39), we construct a MAJSAT problem: whether the majority of the assignments for $x_{0},x_{1},\ldots ,x_{n+1}$ satisfy F=1. In other words, we determine whether the following inequality holds:

(40) $$z+♯k\text{-}IS\geq 2^{n+1}/2=2^{n},$$

The LQC circuit illustrated in Figure 8, with n+1 work qubits, can be used to solve this problem. As mentioned above, the time complexity of this algorithm is $O(n^{4})$.

The detailed procedure is as follows. For a given *k*, we initiate the process by setting $z=2^{n-1}$, denoted as z=100…0 with n−1 zeros, and formulate the Boolean expression $g_{2^{n-1}}$ to determine whether ♯k-IS$+2^{n-1}\geq 2^{n}$ holds. If the result is negative, we keep the first 1 and change the first 0 to 1, i.e., set z=1100…0; if positive, we change the first 1 to 0 and set z=0100…0. This process is then iterated to determine the subsequent numbers in the binary representation of *z*. The iteration continues until the minimum number *z* satisfying Equation (40) is obtained, denoted as $z_{\mathrm{MIN}}$. The value of ♯k-IS is then calculated as $2^{n}-z_{\mathrm{MIN}}$. The time complexity of this search is $O\left(n\right)\cdot O\left(n^{4}\right)=O\left(n^{5}\right)$.

After applying this iterative process for 0-IS, 1-IS, ⋯, up to *n*-IS, we have the solution for MAX-*k*-IS. The total time complexity is $O(n^{6})$, which is polynomial. It is important to note once again that the length of the input for the graph G(n,m) is not *n* but $n^{2}$.

The MAX-*k*-IS problem can be readily reformulated as a decision problem. In this formulation, the input takes the form “G(n,m),k”, where 1≤k≤n. The algorithm accepts the input if the number of independent sets with exactly *k* vertices is the largest compared to the number of independent sets with 1,2,…,n vertices; otherwise, it rejects the input. Thus, $P^{♯P}$ can be seen as a set of decision problems, which can be effectively addressed using an LQC algorithm. In any case, this decision can be implemented by an additional oracle, with the additional oracle qubit serving as the “Y qubit”, as indicated in Equation (15).

The above result implies that the class $P^{♯P}$ is a subset of BLQP. The entire class PH is defined as $\Delta_{0}=\Sigma_{0}=\Pi_{0}=P$; $\Delta_{i}=P^{\Sigma_{i-1}},\Sigma_{i}={\mathrm{NP}}^{\Sigma_{i-1}}$, $\Pi_{i}={\mathrm{co}\text{-}\mathrm{NP}}^{\Sigma_{i-1}}$. According to Toda’s theorem [19], $\mathrm{PH}\subseteq P^{♯P}$. So, the class PH is a subset of BLQP.

### 4.3. BLQP ⊆ $P^{♯P}$

Following the proof of BQP⊆$P^{♯P}$ [20], with some modifications, we now proceed to prove BLQP⊆$P^{♯P}$. Consider a polynomially sized LQC algorithm made of a sequence of logical gates $L_{1}$, $L_{2}$, …, $L_{t}$. Here, t=p(n), with p(n) denoting a polynomial function of the length *n* of the input.

#### 4.3.1. Proof of BLQP⊆$P^{♯P}$ from the Perspective of Computational Problem

In fact, we can prove that BLQP⊆$P^{♯P}$ using various methods. First, suppose there is an algorithm that can obtain a solution encoded by the state $\left|\mathrm{sol})=\right|o_{1}o_{2},\ldots ,o_{n}\rangle ⊗|00\ldots 0)$ with probability P≃1, i.e.,

(41) $$c_{1}e^{i\theta }|o_{1}o_{2}\ldots o_{n}\rangle ⊗|00\ldots 0)+c_{\mathrm{uo}}\left|\mathrm{uo}\right)≃L_{t}L_{t-1}\ldots L_{1}\left|x\right),$$

where |x)=|x〉⊗|00…0) denotes the initial overall state with *n* qubits and q(n) hybits, where q(n) is at most a polynomial function of *n* and θ an arbitrary phase. The state |00…0) indicates that all the hybits are in the |0) state (In the context of the LQC discussed in Section 2, the state becomes unobservable if any hybit is in the |1) state). State |uo) represents a superposition of states, where in each state, at least one hybit is in the |1) state. Thus, the initial state and a meaningful final state encoding the output should have the form |…)=|…〉⊗|00…0). In contrast, intermediate states and non-meaningful final states do not need to adhere to this constraint. Following the convention outlined in Section 2, the notation |) is used for an indefinite inner product state, as the hybits are included in the overall state. Here, $|o_{i}\rangle$ represents the state of the *i*th qubit, where $o_{i}$ can be either 0 or 1. Our algorithm for solving the MIS problem is an example of such an algorithm.

We will next demonstrate that this algorithm can be implemented by a polynomial-time deterministic algorithm equipped with a ♯P oracle. To achieve this, we need to show how to efficiently compute the following amplitudes on a classical computer equipped with a ♯P oracle,

(42) $${\left|A\left(y\right)\right|}^{2}=\left(x\right|L_{1}^{†}L_{2}^{†}\ldots L_{t}^{†}\left|y)(y\right|L_{t}L_{t-1}\ldots L_{1}\left|x\right),$$

where |y) is one of the possible outputs (right for |sol) or wrong for all others). As any state with |1) is unobservable, we only need consider |y) of the following form

(43) |y)=|y〉⊗|00…0).

The amplitude *A* can be decomposed as

(44) $${\left|A\left(y\right)\right|}^{2}=\sum_{z_{1},z_{2},z_{2t-2}}\left(x\right|L_{1}^{†}|z_{2t-2})\ldots (z_{t}|L_{t}^{†}\left|y)(y\right|L_{t}|z_{t-1})\ldots (z_{1}|L_{1}\left|x\right)\;,$$

where the summation is over all possible $z_{i}$s. It is important to note that, in the computational basis, we still have $\sum_{i}|z_{i})(z_{i}|=1$.

Computing any of the terms in the summation (44) is a polynomially sized task, as t=p(n) is a polynomial function of *n*, and each $L_{i}$, in its matrix form, has only a finite number of non-zero elements. This allows us to introduce a language, denoted by +r-DTM, defined by a number *c*. The input is $\left\{|y\right),|z_{1}),\ldots |z_{2t-2}),|x),L_{1},\ldots ,L_{t},k\}$, with $1\leq k\leq 2^{2^{c}}$ being an integer. It is clear that the total length of the input is a polynomial function of *n*. The task is to deterministically calculate

(45) $$a=\left(x\right|L_{1}^{†}|z_{2t-2})\ldots (z_{t}|L_{t}^{†}\left|y)(y\right|L_{t}|z_{t-1})\ldots (z_{1}|L_{1}\left|x\right)$$

and then decide whether Re(a)>0 and k<Re(a). If this is true, the output of the +r-DTM is M=1 (accept); otherwise, M=0 (reject). This is a P problem. Following the proof of BQP⊆$P^{♯P}$ [20], by counting how many times M=1, one can efficiently compute the positive real part of the following summation,

(46) $$|A_{s}{|}^{2}=\sum_{y\in s}{\left|A\left(y\right)\right|}^{2}\;,$$

where *s* is a subset of all $2^{n}$ possible values of *y* associated with the *n* qubits. For example, s={00⋯01,10⋯01}, and $s={\left\{0,1\right\}}^{n}$. Similarly, we can compute efficiently the negative real part and finally $|A_{s}{|}^{2}$.

At this stage, concerns may arise regarding the precision of *a* in Equation (45), which, in the algorithm described above, is of the order O(1). However, this is not problematic because (i) the precision can be easily adjusted, and (ii) for the powerful LQC algorithm, the contribution to the amplitude is exponentially significant relative to the input length *n*. Therefore, a precision of order O(1) suffices. The parameter *c* is chosen based on the specific problem instance, specifically the maximum value *k* in the TM’s input, where $2^{2^{c}}$ exceeds the maximum output |a| of the TM. For sufficiently large *n*, we typically set c=n, ensuring that $2^{2^{n}}$ is significantly larger than both the maximum output |a| of the TM and consequently the total amplitude $|A_{s}{|}^{2}$, which typically scales as exp(n) for the LQC algorithm.

We first compute $|A_{s}{|}^{2}$ for $s={\left\{0,1\right\}}^{n}$ and denote the result as $W_{0}$. This is the total amplitude, which is one for BQP. However, for BLQP, this is usually a large number. Next, we represent an element in *s* that denotes the qubits’ states as an *n*-digit binary number. We then compute $|A_{s}{|}^{2}$ for $s=\{y|y<2^{n-1}\}$ and denote it as $W_{1}$. If $W_{1}/W_{0}≲\varepsilon$, where ε is a small number decreasing exponentially with *n*, and the symbol “≲” means “less than” and “almost equal to”, then we compute $|A_{s}{|}^{2}$ for $s=\{y|2^{n-1}\leq y<3\times 2^{n-1}\}$; otherwise, we compute $|A_{s}{|}^{2}$ for $s=\{y|y<2^{n-2}\}$. In either case, we denote the result as $W_{2}$. If $W_{2}/W_{0}≲1$, then we cut the current interval into half and compute $|A_{s}{|}^{2}$ for one of the halves; otherwise, we cut the other interval into half and compute $|A_{s}{|}^{2}$ for one of the halves. In either case, we denote the result as $W_{3}$, and so on. We continue this for *n* steps and will eventually arrive at a *y* for which ${\left|A\left(y\right)\right|}^{2}∼W_{0}$. This *y* is the solution $|o_{1}o_{2}\ldots o_{n}\rangle ⊗|00\ldots 0)$.

Clearly, this branching algorithm can be extended to cases with polynomially many solutions:

(47) $$c_{1}|{\mathrm{sol}}_{1})+c_{2}|{\mathrm{sol}}_{2})+\ldots +c_{\mathrm{uo}}\left|\mathrm{uo}\right)≃L_{t}L_{t-1}\ldots L_{1}\left|x\right),$$

where $|c_{1}\left|≃\right|c_{2}|≃\ldots$. In this case, we only need to perform polynomial branching to find all the solutions.

So far, we have proven that if a Lorentz quantum circuit can generate a specific state or a superposition of polynomially many states that satisfy the conditions given by the problem, by designing the sequence of gates $L_{1},L_{2},\ldots ,L_{t}$, then it can be implemented by a polynomial-time deterministic algorithm with a ♯P oracle.

#### 4.3.2. Proof of BLQP⊆$P^{♯P}$ by Decision Problem

Since in computational complexity theory any problem is defined as a decision problem, we now examine the decision problem to provide a more formal proof that BLQP⊆$P^{♯P}$.

For a decision problem where the criterion for an accepting state is encoded in a “Y qubit” in the context of BLQP defined in Section 2 (refer to Equation (15)), it is convenient to introduce a projection operator $P_{\mathrm{yes}}$,

(48) $$P_{\mathrm{yes}}=\left[\sum_{j}|j_{Q_{1}}\rangle \langle j_{Q_{1}}|⊗\ldots ⊗\sum_{j}|j_{Q_{n-1}}\rangle \langle j_{Q_{n-1}}|\right]⊗|1_{Y}\rangle \langle 1_{Y}|⊗\left[|0_{H_{1}})(0_{H_{1}}\left|⊗\ldots ⊗\right|0_{H_{q(n)}})(0_{H_{q(n)}}|\right],$$

where *n* is the number of qubits in the current LQC and q(n) the number of hybits, $|\ldots_{Y}\rangle$​​ denotes the state of the “Y qubit”, $|\ldots_{Qi}\rangle$​​ denotes the state of the *i*th qubit, and $|\ldots_{Hi})$​​ denotes the state of the *i*th hybit.

With the projection operator $P_{\mathrm{yes}}$, we have

(49) $$|c_{\mathrm{yes}}{|}^{2}=\left(x\right|L_{1}^{†}L_{2}^{†}\ldots L_{t}^{†}|P_{\mathrm{yes}}|L_{t}L_{t-1}\ldots L_{1}\left|x\right),$$

where $|c_{\mathrm{yes}}{|}^{2}$ is precisely as shown in Equation (15).

In the discussion regarding the relationship between BLQP and $P^{♯P}$ ​​, the amplitude $|c_{\mathrm{yes}}{|}^{2}$ in Equation (49) is decomposed as follows:

(50) $$\begin{array}{ccc} |c_{\mathrm{yes}}{|}^{2} & = & \sum_{z_{1},z_{2},\ldots ,z_{2t}}\left(x\right|L_{1}^{†}|z_{2t})(z_{2t}|L_{2}^{†}|z_{2t-1})\ldots (z_{t+2}|L_{t}^{†}|z_{t+1})(z_{t+1}|P_{\mathrm{yes}}\left|z_{t}\right)\\ & & (z_{t}|L_{t}|z_{t-1})\ldots (z_{2}|L_{2}|z_{1})(z_{1}|L_{1}\left|x\right), \end{array}$$

where each $|z_{i})$ represents a complete orthonormal basis vector, specified within the computational basis.

We introduce a language within the class P, determined by +r-DTM, defined by a number *c*. The inputs are in the form ($|z_{1}),\ldots |z_{2t}),|x\rangle ,L_{1},\ldots ,L_{t},k$), where $1\leq k\leq 2^{2^{c}}$ is an integer for each input, and the total length of the input is a linear function of p(n), thus remaining polynomial in *n*, since p(n) is a polynomial function of *n*.

Given an input, we deterministically calculate

(51) $$a=\left(x\right|L_{1}^{†}|z_{2t})(z_{2t}|L_{2}^{†}|z_{2t-1})\ldots (z_{t+2}|L_{t}^{†}|z_{t+1})(z_{t+1}|P_{\mathrm{yes}}|z_{t})(z_{t}|L_{t}|z_{t-1})\ldots (z_{2}|L_{2}|z_{1})(z_{1}|L_{1}\left|x\right).$$

If Re(a)>0 and k<Re(a), the +r-DTM outputs M=1 (accept); otherwise, it outputs M=0 (reject). Each gate $L_{i}$ is associated with only a few bits. Although $P_{\mathrm{yes}}$​​, as shown in Equation (48), contains an exponential number of elements in the computational basis, calculating $\left(z_{t+1}|P_{\mathrm{yes}}|z_{t}\right)$ is straightforward and thus takes only a single step. Therefore, determining the output of the +r-DTM as M=1 or 0 is a problem within class P when considering the length *n* of the input to the LQC.

If we have access to a ♯P-oracle, which can inform us of the number of accepted paths, with each path representing a problem within class P, we can query this oracle to determine how many times M=1 out of all possible inputs of ($|z_{1}),\ldots |z_{2t}),|x),L_{1},\ldots ,L_{t},k$). This number essentially represents the real positive portion of the following sum:

(52) $$\sum_{z_{1},z_{2},\ldots ,z_{2t}}\left(x\right|L_{1}^{†}|z_{2t})(z_{2t}|L_{2}^{†}|z_{2t-1})\ldots (z_{t+2}|L_{t}^{†}|z_{t+1})(z_{t+1}|P_{\mathrm{yes}}|z_{t})(z_{t}|L_{t}|z_{t-1})\ldots (z_{2}|L_{2}|z_{1})(z_{1}|L_{1}\left|x\right).$$

Thus, by utilizing a ♯P-oracle, we can directly obtain the positive real part of the final amplitude of a Lorentz quantum circuit. Similarly, we define −r-DTM and finally obtain $|c_{\mathrm{yes}}{|}^{2}$.

To accurately compute the acceptance probability in the LQC algorithm, it is crucial to determine the total effective amplitude. This is not simply the identity “1”, but rather the total “observable probability”, given by $|c_{\mathrm{yes}}{|}^{2}+{\left|c_{\mathrm{no}}\right|}^{2}$. This refers to the observable portion of the final state, where all hybits are in the state |0) (see Equation (15) for details). This quantity corresponds to $W_{0}$ in the branching algorithm described earlier. Alternatively, it can be obtained similarly to $|c_{\mathrm{yes}}{|}^{2}$ using a slightly different projection operator $P_{\mathrm{yn}}$ instead of $P_{\mathrm{yes}}$:

(53) $$P_{\mathrm{yn}}=\left[\sum_{j}|j_{Q_{1}}\rangle \langle j_{Q_{1}}|⊗\ldots ⊗\sum_{j}|j_{Q_{n-1}}\rangle \langle j_{Q_{n-1}}|\right]⊗\sum_{j}|j_{Y}\rangle \langle j_{Y}|⊗\left[|0_{H_{1}})(0_{H_{1}}\left|⊗\ldots ⊗\right|0_{H_{q(n)}})(0_{H_{q(n)}}|\right].$$

The total amplitude can then be expressed as

(54) $$\begin{array}{ccc} |c_{\mathrm{yes}}{|}^{2}+{\left|c_{\mathrm{no}}\right|}^{2} & = & \sum_{z_{1},z_{2},\ldots ,z_{2t}}\left(x\right|L_{1}^{†}|z_{2t})(z_{2t}|L_{2}^{†}|z_{2t-1})\ldots (z_{t+2}|L_{t}^{†}\left|z_{t+1}\right)\\ & & (z_{t+1}|P_{\mathrm{yn}}|z_{t})(z_{t}|L_{t}|z_{t-1})\ldots (z_{2}|L_{2}|z_{1})(z_{1}|L_{1}\left|x\right). \end{array}$$

This quantity can be obtained using the same method by querying the ♯P-oracle.

The probability of accepting the input in the LQC circuit is then calculated as $\frac{|c_{\mathrm{yes}}{|}^{2}}{|c_{\mathrm{yes}}{|}^{2}+{\left|c_{\mathrm{no}}\right|}^{2}}$​​. The $P^{♯P}$ algorithm outputs 1 (accept) if $\frac{|c_{\mathrm{yes}}{|}^{2}}{|c_{\mathrm{yes}}{|}^{2}+{\left|c_{\mathrm{no}}\right|}^{2}}\geq 2/3$ and 0 (reject) if $\frac{|c_{\mathrm{yes}}{|}^{2}}{|c_{\mathrm{yes}}{|}^{2}+{\left|c_{\mathrm{no}}\right|}^{2}}\leq 1/3$. Thus, we have shown that BLQP⊆$P^{♯P}$, meaning that if an input can be decided by some BLQP algorithm, it can also be decided by a $P^{♯P}$ algorithm.

Given that we previously established $P^{♯P}$⊆BLQP, we conclude that BLQP=$P^{♯P}$.

We can also generalize the proof for BQP ⊆ PSPACE [1,12] to prove BLQP ⊆ PSPACE. This generalization is rather trivial and straightforward, as the proof for BQP ⊆ PSPACE does not require unitarity for the gate transformation. We have not found an efficient LQC algorithm for the problem of quantified Boolean formulas (QBF), recognized as PSPACE-complete [2,21].

For quantum computing with postselection, it was established that $\mathrm{PostBQP}={\mathrm{BQP}}_{\|,\mathrm{classical}}^{\mathrm{PostBQP}}$ [4]. Similarly, we have $\mathrm{BLQP}={\mathrm{BQP}}_{\|,\mathrm{classical}}^{\mathrm{BLQP}}$, which will be elaborated upon in the following section. As BLQP=$P^{♯P}$, we can directly conclude that

(55) $$P^{♯P}\;={\mathrm{BQP}}_{\|,\mathrm{classical}}^{P^{♯P}\;}.$$

More interesting results are expected for the class $P^{♯P}$ in light of the new perspective provided by BLQP=$P^{♯P}$.

## 5. Comparison Between LQC and Quantum Computing with Postselection

**Overview.** Quantum computing with postselection assumes the ability to discard all computation runs that do not yield a specific measurement outcome, effectively projecting the state onto the desired subspace. LQC, through the CV gate, can simulate this postselection in polynomial time, even when the target subspace has an exponentially small amplitude. However, the LQC also possesses a distinct capability via the CCV gate, which we term “super-postselection.” This allows the LQC to select states based on relative amplitudes (e.g., the number of |1〉s in a basis state) rather than a predetermined yes/no criterion. While postselection can be physically approximated through repeated measurements (requiring exponential time in the worst case), LQC’s super-postselection is achieved deterministically through Lorentz gates, offering a polynomial-time advantage.

As far as we know, the term “postselection” has several meanings. Quite often it refers to a method of selectively choosing specific outcomes after many rounds of quantum measurements [22,23]. The postselection that we are discussing here was introduced by Aaronson as “the power of discarding all runs of a computation in which a given event does not occur” [4]. In other words, it is a ability to force specific outcomes in a single run of quantum measurement, which is beyond quantum mechanics. A quantum computer with this ability of postselection has been found to be very powerful, and the corresponding computational complexity class PostBQP was shown to be equivalent to PP [4]. Below, we briefly review this concept and then discuss the relationship between PostBQP and BLQP.

### 5.1. Simulation of Postselection by LQC

The postselection introduced in Ref. [4] is the ability to efficiently collapse a quantum state given by

(56) $$|\Psi \rangle =\sum_{i}c_{i}|\psi_{i}\rangle =\sum_{j\in \mathrm{yes}}c_{j}|\psi_{j}\rangle +\sum_{k\in \mathrm{no}}c_{k}|\psi_{k}\rangle ,$$

to the following target state,

(57) $$|\Psi_{\mathrm{yes}}\rangle =\frac{1}{\sum_{j\in \mathrm{yes}}{\left|c_{j}\right|}^{2}}\sum_{j\in \mathrm{yes}}c_{j}|\psi_{j}\rangle .$$

Here |Ψ〉 represents a general quantum state, and $|\psi_{i}\rangle$s are basis states that are categorized into `yes’ and `no’ according to a given problem.

Basically, the postselection consists of two operations. The first one behaves like an oracle, marking each state as ’yes’ or ’no’. The second is quantum measurement with the ability to collapse to only yes states. Both of the operations can be simulated by an LQC: the first one with an oracle qubit and the second one with Lorentz transformations on a hybit. The states |Ψ〉 and $|\Psi_{\mathrm{yes}}\rangle$ are stored in *n* work qubits.

The specific process unfolds as follows. The initial state is prepared as

(58) $$|\Psi_{i})=|0_{o}\rangle ⊗|\Psi \rangle ⊗|0).$$

Here, $|0_{o}\rangle$ is the state of the oracle qubit and |0) is for the hybit, which can only undergo Lorentz transformation in the space spanned by |0) and |1). After the oracle operation, which is described in Equation (27) and illustrated in the small box marked “oracle” in Figure 7, the state of the system becomes

(59) $$\begin{array}{c} |\Psi_{o})=|1_{o}\rangle ⊗\sum_{j\in \mathrm{yes}}c_{j}|\psi_{j}\rangle ⊗|0)+|0_{o}\rangle ⊗\sum_{k\in \mathrm{no}}c_{k}|\psi_{k}\rangle ⊗|0)\;, \end{array}$$

where the ’yes’ states and ’no’ states are marked out with the oracle qubit. Within the oracle, whether a given state $|\psi_{i}\rangle$ belongs to ’yes’ or ’no’ can be verified within polynomial time. This oracle operation can be implemented with a conventional quantum computer, and the states in superposition are checked in parallel.

We then use a manipulation that is unique in the LQC. It is the CV gate shown in Figure 4, with the oracle qubit as the control and the hybit as the target. When the oracle qubit is in the state of |1〉, a Lorentz transformation *V* in Equation (8) is applied to the hybit. After applying the CV gate *r* times, we have

(60) $$\begin{array}{cc} |\Psi_{V})= & |1_{o}\rangle ⊗\sum_{j\in \mathrm{yes}}c_{j}\cosh\left(r\chi \right)|\psi_{j}\rangle ⊗|0)+|0_{o}\rangle ⊗\sum_{k\in \mathrm{no}}c_{k}|\psi_{k}\rangle ⊗|0)\\ + & |1_{o}\rangle ⊗\sum_{j\in \mathrm{yes}}c_{j}i\sinh\left(r\chi \right)|\psi_{j}\rangle ⊗|1)\;. \end{array}$$

Because state |1) for the hybit is unobservable, the resulting state is equivalent to

(61) $$\begin{array}{c} |\Psi_{V})=|1_{o}\rangle ⊗\cosh\left(r\chi \right)\sum_{j\in \mathrm{yes}}c_{j}|\psi_{j}\rangle ⊗|0)+|0_{o}\rangle ⊗\sum_{k\in \mathrm{no}}c_{k}|\psi_{k}\rangle ⊗|0)\;. \end{array}$$

where the normalization constant is omitted. If the repetition time is $r\approx \frac{1}{\chi }\ln2^{n}∼O\left(n\right)$, the amplitude for the ’yes’ states is exponentially larger. If the oracle bit is measured, it is almost certain to find it in the state of $|1_{o}\rangle$. Thus, after the measurement, we are almost certain to find the system in the following state

(62) $$|\Psi_{f})=|1_{o}\rangle ⊗\sum_{j\in \mathrm{yes}}c_{j}|\psi_{j}\rangle ⊗|0).$$

The postselection is accomplished.

It is apparent that even when the absolute values of $|c_{j}|$s are very different, e.g., some $|c_{j}|$s are exponentially smaller than the others, we can still obtain the states (62) in polynomial time in *n*.

### 5.2. Super-Postselection by LQC

In Section 3, we have discussed an operation called *Q*, whose LQC circuit is shown in Figure 6. The *Q* operation is capable of counting the number of qubits in state |1〉 within $|\psi_{i}\rangle$ and then uses this count to appropriately amplify the amplitude. By repeating *Q* sufficient number of times, one can select the basis states that have the largest number of qubits in |1〉.

Consider, for example, a superposition state given by

(63) $$|\Phi_{0})=\left[|1000\rangle +|0110\rangle \right]⊗|1_{o}\rangle ⊗\left|0\right),$$

which is not normalized, because the normalization is not important. We apply *Q* with four work qubits to this state *r* times, and the state becomes

(64) $$\begin{array}{cc} |\Phi_{r})= & \left[\cosh(r\chi )|1000\rangle +\cosh(2r\chi )|0110\rangle \right]⊗|1_{o}\rangle ⊗\left|0\right)\\ + & i\left[\sinh(r\chi )|1000\rangle +\sinh(2r\chi )|0110\rangle \right]⊗|1_{o}\rangle ⊗\left|1\right)\;. \end{array}$$

For $\chi =4\ln(\sqrt{2}+1)$, when r=4, the ratio between the coefficient before |1000〉 and the coefficient before |0110〉 is over $10^{6}$. Because |1) is unobservable, the above state can be regarded approximately as

(65) $$|\Phi_{r})\approx |0110\rangle ⊗|1_{o}\rangle ⊗|0)\;.$$

The state |0110〉 is selected. However, let us start with a different superposition state

(66) $$|\Phi_{0})=\left[|1110\rangle +|0110\rangle \right]⊗|1_{o}\rangle ⊗\left|0\right)\;.$$

This time we do the same *Q* operation four times. What is selected at the end is |1110〉 instead of |0110〉, because |1110〉 has more ones than |0110〉.

From the above example, it is clear that the selection achieved by repeated *Q* operations is relative. This is in stark contrast to postselection, which is done according to a preset criterion. It is precisely due to this special selection capability of LQC that we are able to solve the MIS problem in polynomial time with the circuit shown in Figure 7. We call the selection by *Q* super-postselection just to distinguish it from Aaronson’s postselection introduced in Ref. [4].

The above comparison shows that an LQC can efficiently solve any problem that can be efficiently solved by a quantum computer with postselection. However, the reverse is not necessarily true due to the super-postselection capability of LQC. This implies that the complexity class PostBQP is a subset of BLQP, but it may not be a strict subset. The reason for this is that a problem that an LQC solves with super-postselection may be solved efficiently by postselection using a different strategy.

### 5.3. A Relation Analogous to That of PostBQP

As $\mathrm{BQP}={\mathrm{BQP}}^{\mathrm{BQP}}$ [20], we would expect $\mathrm{BLQP}={\mathrm{BLQP}}^{\mathrm{BLQP}}$. However, upon closer examination, one realizes that this expectation may be incorrect because, in an LQC, any non-zero error probability can be arbitrarily magnified by Lorentz gates. Instead, as discussed in Ref. [4], we generally have $\mathrm{BLQP}={\mathrm{BQP}}_{\|,\mathrm{classical}}^{\mathrm{BLQP}}$​​, which represents the class of problems solvable by a machine capable of making a polynomial number of non-adaptive classical queries to a BLQP oracle, followed by a polynomial-time quantum computation. The requirement that the queries be non-adaptive is further explained as follows.

Consider a query to a BLQP oracle that, without loss of generality, yields a result denoted as follows:

(67) $$c_{O}|O\rangle +c_{\mathrm{error}}|\mathrm{error}\rangle .$$

Here, |O〉 represents the expected correct result produced by the oracle, while |error〉 represents an irrelevant outcome, an inherent feature of any bounded-error algorithm, characterized by a very small amplitude $c_{\mathrm{error}}$. The subsequent gates are designed to produce the expected result assuming the error is zero. Thus, without loss of generality, the output can be expressed as follows:

(68) $$c_{O}U|O\rangle +c_{\mathrm{error}}U|\mathrm{error}\rangle =c_{O}|\mathrm{expected}\rangle +c_{\mathrm{error}}U|\mathrm{error}\rangle ,$$

where |expected〉 is the final state that we anticipate when we design the algorithm.

In conventional quantum algorithms, the unitarity of the operator *U* ensures no magnification of small $|c_{\mathrm{error}}{|}^{2}$. Consequently, adaptive queries to a BQP oracle are feasible in conventional quantum computers, resulting in $\mathrm{BQP}={\mathrm{BQP}}^{\mathrm{BQP}}$. However, in the context of an LQC, the operator *U* can be Lorentzian, potentially leading to an amplification of the second term in Equation (68), even when $|c_{\mathrm{error}}{|}^{2}$ is exponentially small. As a consequence, queries to BLQP oracles in LQC must be non-adaptive, implying independence or parallelism. The primary circuit managing the results of each oracle can only contain unitary gates, resulting in $\mathrm{BLQP}={\mathrm{BQP}}_{\|,\mathrm{classical}}^{\mathrm{BLQP}}$. This is the same reasoning behind $\mathrm{PostBQP}={\mathrm{BQP}}_{\|,\mathrm{classical}}^{\mathrm{PostBQP}}$ [4].

However, if a measurement is taken immediately after each query to the BLQP oracle, such that

(69) $$c_{O}|O\rangle +c_{\mathrm{error}}|\mathrm{error}\rangle \overset{\mathrm{measurement}}{\to }|O\rangle ,$$

then the exponentially small error vanishes and is not amplified by the next gate. Consider the scenario where we make polynomial number *p* of queries to BLQP oracles, each with an exponentially small error probability of $c^{-p}$ (c>1). In this scenario, regardless of whether the queries are adaptive or non-adaptive, the probability of getting the correct result for the entire circuit is given by

(70) $$P={\left(1-c^{-p}\right)}^{p}\to 1.$$

Since the time for each measurement is usually regarded as “1”, polynomial measurements are allowed for a polynomial algorithm. With the inclusion of post-measurement operations, we have $\mathrm{BLQP}=P^{\mathrm{BLQP}}$, since “P” denotes classical polynomial-time computation, and each step of a classical algorithm can only access a definite outcome determined by a measurement.

## 6. Summary

In summary, we have demonstrated the superior power of the Lorentz quantum computer (LQC) through concrete examples. These results show that its computational complexity class BLQP (bounded-error Lorentz quantum polynomial-time) is equivalent to $P^{♯P}$. In comparison, it is not even clear whether the complexity class BQP associated with the conventional quantum computer contains NP or not. Our work will likely motivate further study into the LQC to better understand its capabilities.

This work also reveals a fascinating relation between computational power and physics. In Ref. [4], it is argued that quantum mechanics is an island in the “theoryspace”. The LQC appears to add an intriguing twist on this claim. On the one hand, Lorentz quantum mechanics seems drastically different from quantum mechanics by having unobservable states while living in an indefinite inner product space with complex Lorentz transformations [9,10]. On the other hand, the Bogoliubov exications, quasi-particles of bosonic many-body systems, do behave approximately like a Lorentz quantum mechanical system [6].

Although the physical realization of hybits remains an open challenge, the theoretical framework of the LQC offers profound insights into the interplay between computational complexity and physical principles. Our results suggest that even idealized extensions of quantum mechanics, such as those with indefinite inner products, can dramatically expand the landscape of efficient computation.

## Data Availability

The original contributions presented in this study are included in the article. Further inquiries can be directed to the corresponding author.

## Appendix A. State of the Oracle Qubit, the Auxiliary Qubit, and the Hybit

After step (iv) of the algorithm solving MAJSAT, we have (dropping the state for work qubits |11…1〉)

(A1) $$|\psi_{\mathrm{iv}})=|\phi_{o}\rangle ⊗|0)⊗|\varphi_{\beta /\alpha }\rangle ,$$

where $|\phi_{o}\rangle =a_{s}|0_{o}\rangle +b_{s}|1_{o}\rangle$ with

(A2) $$a_{s}=\frac{N-s}{\sqrt{{\left(N-s\right)}^{2}+s^{2}}}\;,\;\;\;b_{s}=\frac{s}{\sqrt{{\left(N-s\right)}^{2}+s^{2}}}\;.$$

After applying the controlled-Hadamard gate at the step (v), we have

(A3) $$\begin{array}{cc} |\psi_{v}) & =\alpha |\phi_{o}\rangle ⊗\left|0\right)⊗|0\rangle +\frac{\beta }{\sqrt{2}}\left[\left(a_{s}+b_{s}\right)|0_{o}\rangle +\left(a_{s}-b_{s}\right)|1_{o}\rangle \right]⊗\left|0\right)⊗|1\rangle \\ \; & =|0_{o}\rangle ⊗\left|0\right)⊗\left[\alpha a_{s}|0\rangle +\frac{\beta (a_{s}+b_{s})}{\sqrt{2}}|1\rangle \right]+|1_{o}\rangle ⊗\left|0\right)⊗\left[\alpha b_{s}|0\rangle +\frac{\beta (a_{s}-b_{s})}{\sqrt{2}}|1\rangle \right] \end{array}$$

After the application of CV gate $r^{\prime }$ time at the step (vi) and the omission of terms that are either unobservable or exponentially small, this state becomes

(A4) $$|\psi_{\mathrm{vi}})\approx |1_{o}\rangle ⊗|0)⊗\left[\alpha b_{s}|0\rangle +\frac{\beta (a_{s}-b_{s})}{\sqrt{2}}|1\rangle \right]\;.$$

The exact probability of having this state is

(A5) $$P_{\beta /\alpha }=\frac{\cosh^{2}\left(2r\chi \right)}{2^{n}-1+\cosh^{2}\left(2r\chi \right)}\cdot \frac{\cosh^{2}\left(r^{\prime }\chi \right)\left(\alpha^{2}s^{2}+\beta^{2}{\left(2^{n}-2s\right)}^{2}/2\right)}{\cosh^{2}\left(r^{\prime }\chi \right)\left(\alpha^{2}s^{2}+\beta^{2}{\left(2^{n}-2s\right)}^{2}\right)/2+\alpha^{2}{\left(2^{n}-s\right)}^{2}+\beta^{2}2^{2n-1}}\;,$$

where $\chi =2\ln(\sqrt{2}+1)$. We have $P_{\beta /\alpha }\approx 1$ when $r\approx r^{\prime }\approx \ln N/\chi \propto n$.

## Appendix B. Possible Values of *η*=*β*/*α*

The probability of obtaining −1 when the auxiliary qubit state $\varphi_{\eta }$ is measured along the *x*-direction is

(A6) $$\begin{array}{cc} P_{-} & =|\langle -|\varphi_{\eta }\rangle {|}^{2}\\ \; & =\frac{|s-\eta \sqrt{1/2}\left(2^{n}-2s\right){|}^{2}}{2\left[s^{2}+\eta^{2}{\left(2^{n}-2s\right)}^{2}/2\right]}\\ \; & =\frac{s^{2}+\eta^{2}{\left(2^{n}-s\right)}^{2}/2-\sqrt{2}\eta s\left(2^{n}-2s\right)}{2\left[s^{2}+\eta^{2}{\left(2^{n}-2s\right)}^{2}/2\right]}\\ \; & =\frac{1}{2}-\frac{\sqrt{2}\eta s\left(2^{n}-2s\right)}{2s^{2}+\eta^{2}{\left(2^{n}-2s\right)}^{2}} \end{array}$$

When $s>2^{n-1}$, the second term on the right hand side is positive and we re-write it as

(A7) $$P_{-}-\frac{1}{2}=\frac{\sqrt{2}\eta s\left(2s-2^{n}\right)}{2s^{2}+\eta^{2}{\left(2s-2^{n}\right)}^{2}}\;.$$

It can be shown, for a given probability $0<\delta_{p}<1/2$, when

(A8) $$\frac{(1-\sqrt{1-4\delta_{p}^{2}})s}{\sqrt{2}\delta_{p}\left(2s-2^{n}\right)}\leq \eta \leq \frac{(1+\sqrt{1-4\delta_{p}^{2}})s}{\sqrt{2}\delta_{p}\left(2s-2^{n}\right)}\;,$$

we have

(A9) $$P_{-}-\frac{1}{2}\geq \delta_{p}\;.$$

Let $s=2^{n-1}+\delta s$ and we have

(A10) $$\frac{(1-\sqrt{1-4\delta_{p}^{2}})s}{2\sqrt{2}\delta_{p}\;\delta s}\leq \eta \leq \frac{(1+\sqrt{1-4\delta_{p}^{2}})s}{2\sqrt{2}\delta_{p}\;\delta s}\;,$$

When $\delta s=2^{n-1}$ we have the ratio s/δs=2, which is the smallest. This shows that to satisfy the inequality (A9) for a given $\delta_{p}$ for all possible values of *s*, we must have

(A11) $$\eta_{m}-\eta_{s}\geq \frac{2\sqrt{1-4\delta_{p}^{2}}}{\sqrt{2}\delta_{p}}\;,$$

where $\eta_{m}$ and $\eta_{s}$ are the largest and smallest values of η. Since η takes only discrete values in the form of $2^{j}$ with j∈[−n,n], the number of η satisfying the inequality (A9) increases with smaller $\delta_{p}$.

## Appendix C. Boolean Expression for the Function *g_z_*

The Boolean formula $g_{z}\left(x_{1},x_{2},\cdots ,x_{n}\right)$ used in the main text is defined as $g_{z}=1$ if the string $x=x_{1}x_{2}\cdots x_{n}$, interpreted as a binary number, is less than *z*. When x≥z, we have $g_{z}=0$.

We use an example to show how to construct $g_{z}\left(x_{1},x_{2},\ldots ,x_{n}\right)$. Assume that n=8 and z=10101001, we then have

(A12) $$g_{z}={\bar{x}}_{1}\vee \left({\bar{x}}_{2}\wedge {\bar{x}}_{3}\right)\vee \left({\bar{x}}_{2}\wedge {\bar{x}}_{4}\wedge {\bar{x}}_{5}\right)\vee \left({\bar{x}}_{2}\wedge {\bar{x}}_{4}\wedge {\bar{x}}_{6}\wedge {\bar{x}}_{7}\wedge {\bar{x}}_{8}\right)\;.$$

The length of the Boolean expression thus constructed is always less than $n^{2}$. This construction method is applicable to different values of *N*.
